# Engineering of *Aspergillus niger* for efficient production of d-xylitol from l-arabinose

**DOI:** 10.1186/s12934-024-02526-7

**Published:** 2024-10-05

**Authors:** Marcel Rüllke, Veronika Schönrock, Kevin Schmitz, Mislav Oreb, Elisabeth Tamayo, J. Philipp Benz

**Affiliations:** 1 Fungal Biotechnology in Wood Science, Holzforschung München, TUM School of Life Sciences, Technical University of Munich, 85354 Freising, Germany; 2 Faculty of Biological Sciences, Institute of Molecular Biosciences, Goethe University Frankfurt, 60438 Frankfurt am Main, Germany

**Keywords:** Xylitol production, *Aspergillus niger*, l-arabinose conversion, Xylitol transporter, Arabinanase production, Carbon catabolite repression

## Abstract

d-Xylitol is a naturally occurring sugar alcohol present in diverse plants that is used as an alternative sweetener based on a sweetness similar to sucrose and several health benefits compared to conventional sugar. However, current industrial methods for d-xylitol production are based on chemical hydrogenation of d-xylose, which is energy-intensive and environmentally harmful. However, efficient conversion of l-arabinose as an additional highly abundant pentose in lignocellulosic materials holds great potential to broaden the range of applicable feedstocks. Both pentoses d-xylose and l-arabinose are converted to d-xylitol as a common metabolic intermediate in the native fungal pentose catabolism.

To engineer a strain capable of accumulating d-xylitol from arabinan-rich agricultural residues, pentose catabolism was stopped in the ascomycete filamentous fungus *Aspergillus niger* at the stage of d-xylitol by knocking out three genes encoding enzymes involved in d-xylitol degradation (Δ*xdhA*, Δ*sdhA*, Δ*xkiA*). Additionally, to facilitate its secretion into the medium, an aquaglyceroporin from *Saccharomyces cerevisiae* was tested. In *S. cerevisiae*, Fps1 is known to passively transport glycerol and is regulated to convey osmotic stress tolerance but also exhibits the ability to transport other polyols such as d-xylitol. Thus, a constitutively open version of this transporter was introduced into *A. niger*, controlled by multiple promoters with varying expression strengths. The strain expressing the transporter under control of the P*tvdA* promoter in the background of the pentose catabolism-deficient triple knock-out yielded the most favorable outcome, producing up to 45% d-xylitol from l-arabinose in culture supernatants, while displaying minimal side effects during osmotic stress. Due to its additional ability to extract d-xylose and l-arabinose from lignocellulosic material via the production of highly active pectinases and hemicellulases, *A. niger* emerges as an ideal candidate cell factory for d-xylitol production from lignocellulosic biomasses rich in both pentoses.

In summary, we are showing for the first time an efficient biosynthesis of d-xylitol from l-arabinose utilizing a filamentous ascomycete fungus. This broadens the potential resources to include also arabinan-rich agricultural waste streams like sugar beet pulp and could thus help to make alternative sweetener production more environmentally friendly and cost-effective.

**Supplementary Information:**

The online version contains supplementary material available at 10.1186/s12934-024-02526-7.

## Background

In agriculture and food value chains, huge amounts of waste are produced, causing logistics and environmental problems. These waste streams nevertheless harbor great potential for producing valuable carbon-derived compounds for various industrial applications. Sugar beet (*Beta vulgaris* subsp. *vulgaris*) is an important agricultural crop mainly used in sucrose production and accounts for almost 16% of global sugar production [1, 2]. In Germany alone, 28.2 million tons of sugar beet were produced in 2022, being this country the world's fourth largest sugar beet producer [1]. After sucrose extraction, the leftover sugar beet pulp is used either for biogas production or delivered as supplementation feed for livestock [3]. In addition to proteins, lipids, and salts, sugar beet pulp contains, among others, the constituent sugars d-glucose (22% w/w), l-arabinose (18% w/w), uronic acids (18% w/w), d-galactose (5% w/w), d-rhamnose (2% w/w), d-xylose (2% w/w), d-mannose (1% w/w), and 4% w/w residual sucrose [4, 5]. Converting these diverse compounds into new products would ensure a more comprehensive utilization of this agricultural crop, with d-xylitol being one notable example [6].

Xylitol is a five-carbon polyol naturally found in trace amounts in fruits, vegetables, fungi, and algae [7]. Unlike low-caloric sweeteners such as saccharin and aspartame, it has similar sweetness, taste, and water solubility to sucrose [2]. Furthermore, it is compatible with diabetic patients due to its insulin-independent metabolism, has half of the caloric content as sucrose, and is beneficial to health [8]. For example, due to its anti-inflammatory potential, it can help to treat diseases of the respiratory tract and middle ear. In addition, because it decreases the growth levels of pathogenic *Streptococcus* sp. at the very early stages, it is being used as a preventive agent for dental caries [9]. These advantages result in numerous applications of d-xylitol in food science, medicine, and personal care products, driving an increasing market value [10].

Currently, there are two main routes for industrial production of d-xylitol: chemical and biotechnological, both based on the conversion of d-xylose from hemicellulosic hydrolysates [11]. Despite the high yield of d-xylitol, the chemical process is very expensive and environmentally unfriendly due to the numerous steps of chemical conversions [12]. Biotechnological d-xylitol production offers several advantages compared to the chemical route, such as no need for d-xylose purification and crystallization and the non-requirement of high pressures and temperatures, as well as ecologically damaging catalysts like Raney-nickel for d-xylose conversion [13]. Nevertheless, the advantages of this route will depend on the sustainability of its lifecycle and the efficient integration of the process in a biorefinery. Current biotechnological production processes for of d-xylitol includes microbial conversion of d-glucose or d-xylose into d-xylitol using yeast, as well as release of d-xylose from xylan [7, 11, 14]. However, the associated production costs are still significant since most yeasts cannot produce sufficient xylanolytic enzymes to retrieve d-xylose directly from complex substrates and the preparation of d-xylose reductase (which reduces d-xylose to d-xylitol) is cumbersome and costly.

**Fig. 1:**
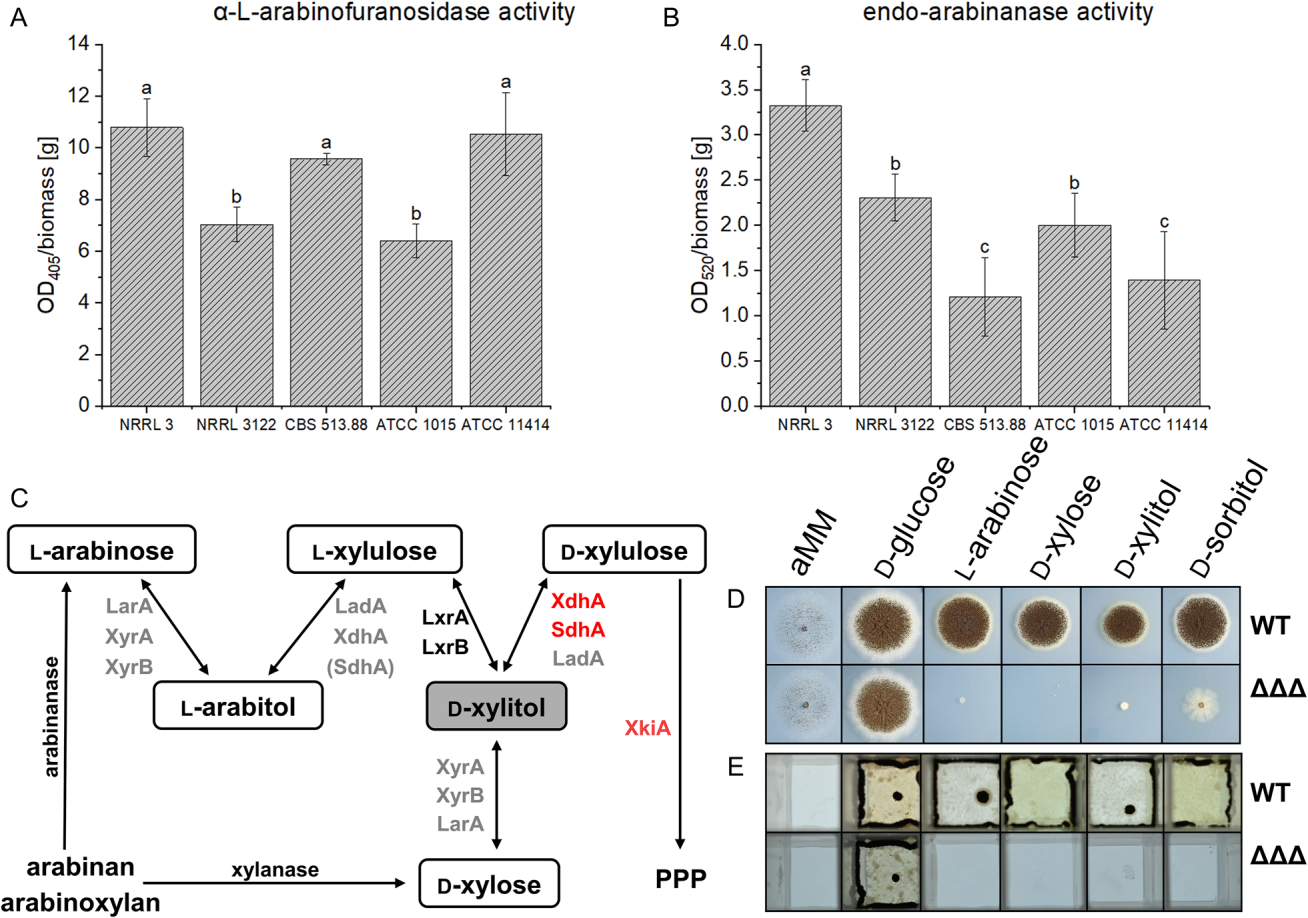
Selection of an *Aspergillus niger* strain for triple KO and growth in different carbon sources. α-L-arabinofuranosidase (**A**) and endo-arabinanase (**B**) production of five different *A. niger* strains were compared in aMM containing 1% pectin, 0.25% xylan, 0.125% arabinan, and 0.5% carboxymethylcellulose sodium salt using colorimetric assays (Megazyme). Data are mean ± standard deviation. Different letters indicate statistically significant differences according to an ANOVA-test (n = 4), p < 0.05. **C** Pathway of arabinan/arabinoxylan catabolism including the enzymes involved in production and consumption of d-xylitol: enzymes for which there is evidence that they have more than one function are depicted in grey, enzymes with unique function in black. For SdhA only a side activity on l-arabitol was reported (brackets). Enzymes that were deleted in the base strain to influence d-xylitol degradation are highlighted in red. PPP, pentose phosphate pathway. The triple knock-out Δ*xdhA,* Δ*sdhA* and Δ*xkiA* (ΔΔΔ) was tested for growth on 20 ml of solid aMM (**D**) and in 3 ml of liquid aMM (**E**) with d-glucose, l-arabinose, d-xylose, d-xylitol and d-sorbitol as carbon sources

Filamentous fungi such as *Aspergillus niger*, *Aspergillus oryzae,* and *Trichoderma reesei* have been studied for d-xylitol production from d-xylose as a substrate [15, 16]. In contrast to yeasts, these fungi can naturally release d-xylose from lignocellulosic biomass and metabolize it to d-xylitol [17]. *A. niger* is capable of producing a wide range of hydrolytic enzymes to degrade complex sugars polymers such as lignocellulose, including xylanases and arabinanases [18]. This fungus is considered an important fungal cell factory, and its natural metabolic capabilities enable the production of several valuable by-products [19]. In *A. niger*, both d-xylose and l-arabinose are natively converted to d-xylitol as a common metabolic intermediate in the pentose catabolic pathway (PCP). Those pentoses are initially reduced by d-xylose reductases (XyrA, XyrB) and l-arabinose reductase (LarA) to d-xylitol and l-arabitol (syn. l-arabinitol), respectively. l-Arabitol is converted to d-xylitol by two additional steps, which is natively further catabolized to d-xylulose by d-xylitol dehydrogenase (XdhA) or two somewhat unspecific side reactions, including l-arabinitol-4-dehydrogenase (LadA) and d-sorbitol dehydrogenase (SdhA) (Fig. 1C). This is followed by the first irreversible step of the pathway, catalyzed by the d-xylulose kinase XkiA, which is also the first dedicated step into the pentose phosphate pathway (PPP) [20, 21, 22]. Recently, *A. niger* strains were engineered to improve d-xylitol production from d-xylose and lignocellulosic biomass by knocking-out several enzymes involved in d-xylitol catabolism [21]. These authors found that the triple mutant Δ*ladA*Δ*xdhA*Δ*sdhA* showed the best performance in d-xylitol production from cottonseed hulls and wheat bran. Furthermore, they showed the involvement of XkiA in the degradation of d-xylulose, a downstream product of the d-xylitol degradation. Nevertheless, the d-xylitol production in this study relied exclusively on d-xylose, whose concentration is relatively low in sugar beet pulp compared to l-arabinose. To the best of our knowledge, a biotransformation pathway from l-arabinose has not been explored or demonstrated in a filamentous fungus. In this work, we, therefore, aimed to genetically engineer *A. niger* for d-xylitol generation from l-arabinose as a strategy to extend the feedstock possibilities to arabinan-rich waste streams and thus to identify more economical and environmentally friendly production routes.

## Methods

### Filamentous fungal strains and culture conditions

All *A. niger* strains used in this work (Supplements) were cultivated in *Aspergillus* Minimal Medium (aMM) containing 2% d-glucose as a carbon source or in *Aspergillus* complete Medium (aCM), which is based on aMM complemented with 2% d-glucose, 0.5% yeast extract and 0.1% casamino acids [23]. For generation of conidia 39 g/l potato extract d-glucose agar (Roth) was added to aCM. After 10 days of cultivation at 30 °C and constant light, the spores were harvested using a 0.89% NaCl and 0.05% Tween 80 solution. 1 mM uridine and 21 µM nicotinamide were added to the medium when needed.

### Cultivation for enzyme assays

To measure the capacity of hydrolase production in various *A. niger* strains, 10^6^ conidia per ml were used to inoculate 50 ml of aMM supplemented with 1% pectin (from citrus peel, Carl Roth GmbH + Co. KG), 0.25% xylan (birchwood; Megazyme), 0.125% arabinan (sugar beet, Megazyme) and 0.5% carboxymethylcellulose sodium salt (Sigma-Aldrich) to induce the production of cellulases and hemicellulases. After 96 h of incubation at 30 °C and 250 revolutions per minute (rpm), the supernatants were used for endo-arabinanase and α-L-arabinofuranosidase activity assays.

### Endo-arabinanase assay

As substrate for the endo-arabinanase assay (Megazyme protocol adapted for microtiter format) a 20 g/l solution of red debranched sugar beet arabinan (NEOGEN; Megazyme) in 200 mM of sodium acetate buffer (pH 4.5) was used. For each sample, 32 µl of the arabinan substrate was mixed with 8 µl of 100 mM sodium acetate buffer (pH 4.5) and 20 µl of ddH_2_O. In an ice-cooled microtiter plate, 60 µl of the mastermix was added into two wells per sample and 10 µl of the enzyme supernatant was added to one well. After incubation at 40 °C for 2 h, the plate was cooled on ice for 2 min and 10 µl of supernatant was added to the second well for normalization. To precipitate the unhydrolyzed substrate 240 µl of 99% ethanol was added. This was followed by 3 min of centrifugation at 4700 × g. 200 µl of the supernatant was added into a transparent microtiter plate with flat bottom to measure the absorbance at 520 nm. The OD values were normalized to the cultures dry weight.

### α-L-arabinofuranosidase assay

To measure α-L-arabinofuranosidase activity 6 µl of 5 mM of nitrophenyl α-l-arabinofuranoside (NEOGEN; Megazyme) in 50% ethanol was mixed with 44 µl of 100 mM sodium acetate buffer (pH 4.5) and 10 µl of ddH_2_0 and added to an ice-cold microtiter plate (two wells per sample). 10 µl of 1:10 diluted enzyme supernatant was added to one of the two wells and incubated by 40 °C for 15 min. The plate was cooled on ice before 10 µl of supernatant was added to the second well. Afterwards, 230 µl of 250 mM of sodium hydroxide solution was added. 200 µl of the reaction mix was transferred into a transparent microtiter plate with flat bottom to measure the absorbance at 405 nm. The OD values were normalized to the cultures dry weight.

### Cloning of the luciferase and FPS1 d-xylitol transporter cassettes

The cloning was executed according to the protocols of Sambrook and Russel [24]. The *S. cerevisiae FPS1* transporter sequence was codon-optimized for *A. niger* using the codon harmonizer software (Horst Lechner, to be published) and synthesized by Eurofins Genomics. A constitutively open variant of the transporter was generated, according to Tamás et al. [25] by deleting the 16 amino acids (aa) 216–231. Three constitutive promoters with different strengths were used to build transporter and luciferase expression cassettes: the two promoters P*tvdA* and P*mbfA*, and a modified version of the P*gpdA* promoter derived from *A. niger*. [26, 27]. The P*gpdA* promoter was amplified from *A. niger* NRRL3 gDNA, and annealing oligos were ligated into the sequence to generate a version with three *gpd* boxes [28]. To test the promoter expression rates, luciferase constructs were generated combining the different promoters with a *Neurospora crassa* codon-optimized *Photinus pyralis* luciferase gene (pXM1.1) (after deleting the peroxisomal targeting sequence) combined with a PEST signal for faster degradation and the t*TrpC* terminator [29–31]. The cassettes containing one of the three constitutive promoters in combination with the constitutively open or the wildtype (WT) variant of the transporter and the *A. nidulans* terminator t*TrpC* were inserted into the multi-cassette backbone by BsmBI-based Golden Gate cloning [32]*.*

### Cloning for the *A. niger* gene KO constructs

To delete the *xdhA, sdhA,* and *xkiA* genes, repair template plasmids were constructed by cloning 1.3–1.5 kb long flanking regions of the coding sequences into the pYTK095 backbone (AddGene) using Gibson assembly. For the disruption of *xdhA*, the repair plasmid was designed according to the established protocol from [20]. NotI restriction sites were added to linearize the construct before the transformation. For double-strand break generation and a higher probability of a KO event, a CRISPR/Cas9 (Clustered Regularly Interspaced Short Palindromic Repeats,CRISPR associated protein 9) plasmid pKS017, with the guide RNA 5′-GAAAGCATCACCCTACAGGA -3′ for the *xdhA* KO, 5′-GCACATGTATCCCTGCAACA -3′ for the *sdhA* KO, 5′-TAGGGGCAGAGCAAGAATGG -3′ for the *xkiA* KO was co-transformed to the linear repair templates [33–35]. CreA KO and truncations were performed according to [35].

### Genetic manipulation of filamentous fungi

Protoplasts of *kusA* deficient *A. niger* strains (Supplementary Table 1) were transformed according to the established protocol of Niu et al. [36]. The transporter and luciferase cassettes were integrated into the truncated *nicB* locus for nicotinamide complementation and integration [37]. The KOs of the d-xylitol catabolic genes were performed in a *kusA* and *pyrG* KO strain by CRISPR/Cas9 based gene deletions using self-replicating plasmids carrying a *pyrG* complementation cassette. Transformant strains were validated by PCR post-extraction of genomic DNA according to an established protocol [33]. Three rounds of singulations were performed for all validated transformants. To eliminate the CRISPR/Cas9-plasmid, KO strains were grown on aMM 1.5% agar plates containing 1 mM uridine and 0.75 mg/ml 5-fluorooratic acid (5-FOA) and homokaryons were obtained by three rounds of singulation [38]. To evaluate the phenotypic effects of the Kos, growth of 2 µl of 10^8^ conidia/ml on 20 ml aMM solid plates supplemented with 1% d-glucose, l-arabinose, d-xylose, d-xylitol and d- sorbitol was assayed. Plates were incubated at 30 °C for 6 d without light.

### Luciferase reporter assay

To test the expression strength of the various promoters, a white 96-well microtiter plate with a transparent bottom and Breathe-Easy^®^ sealing membrane was inoculated with 10^6^ spores/ml containing 200 µl of aMM + 1% of d-fructose (substituting for d-glucose to minimize CCR effects), 1 mM uridine and 1 mM d-luciferin (Cayman chemicals). For incubation at 30 °C, orbital shaking, and tracking luminescence over time, a SPARK plate reader (Tecan) was used. In addition, the OD_600_ was measured to normalize the light emission per biomass.

### Shake flask assay

To generate biomass, 100 ml of aMM containing 1% d-glucose, 1 mM uridine, and 21 µM nicotinamide were inoculated with 10^6^ conidia/ml in 250 ml non-baffled shake-flasks and incubated at 250 rpm and 30 °C. After 99 h, d-glucose was used up and 1% l-arabinose was added (t = 0). A supernatant sample was taken every 24 h starting at 66 h after l-arabinose addition. To test the truncation strains, 100 ml aMM with 1% d-glucose and 1% l-arabinose was inoculated with 10^6^ conidia/ml (t = 0). After 66 h of incubation at 250 rpm and 30 °C, the first supernatant sampling was performed, followed by sampling every 24 h.

### Sugar analysis by HPLC

For the sugar concentration measurement, a system from Shimadzu (CBM-40 system controller, RID-20A refractive index detector, SPD-M40 Photodiode array detector, DGU-405 degassing unit, LC-40D XR solvent delivery module, SIL-40 XR Autosampler, CTO-40S column oven) and a Coregel 801 FA column (Concise Separations) were used. After preheating the oven to 70 °C and purging the system with the mobile phase consisting of 5 mM H_2_SO_4_ and 30% acetonitrile, 10 µl of the sample was injected at the flow rate of 0.4 ml/min.

To calibrate the system, a series of standard dilutions ranging from 1 mg/ml to 10 mg/ml of d-glucose, l-arabinose, and d-xylitol supplemented with 1 mM uridine and 21 µM nicotinamide was measured. LabSolutions software (Shimadzu) was used to determine the concentrations.

### Osmotic stress assay

To test the phenotypic effects of different osmolytes, 10^6^ conidia/ml were inoculated into 3 ml of aMM containing 1% d-glucose, 1 mM uridine and 21 µM nicotinamide and one of the osmolytes NaCl (1–4%), d-sorbitol (20–70%), polyethyleneglycol-6000 (10–30%) or d-xylitol (10–30%) in different concentration ranges. The 24-deep well plates were incubated at 250 rpm and 30 °C for 6 days. To assess growth under salt stress on 20 ml of solid medium plates, aMM containing 1.5% agar was supplemented with 1–4% NaCl and 2 µl of 10^8^ conidia/ml suspension was pipetted in the middle of the plate and incubated at 30 °C for 6 d without light.

### Statistical analyses

Data shown in graphs typically represent the mean of at least three biological replicates for each condition (n ≥ 3) and error bars correspond to the standard deviation (SD). For data comparing two groups, the Shapiro–Wilk test for normality was carried out. In cases where one of the treatments (or both) was not normally distributed, a Mann–Whitney U test was applied. If the data were normally distributed, a two-tailed Student’s *t*-test was applied for the comparison of the conditions. Significance is indicated by asterisks (*p < 0.05; **p < 0.01).

## Results

### Comparison of α-L-arabinofuranosidase and endo-arabinanase production of different commonly used *A. niger* strains

To generate a strain able to produce d-xylitol from arabinan-containing feedstocks, a strain harboring the potential to produce large amounts of arabinanases would be favorable. Hence, five different *A. niger* strains were compared for their production of endo-arabinanase and α-L-arabinofuranosidase in aMM containing arabinan and other polysaccharides. The highest α-L-arabinofuranosidase activities normalized to the specific dry mass were measured for the strains NRRL3, CBS 513.88, and ATCC 11414 (Fig. 1A). NRRL3 outperformed the other strains in the endo-arabinanase activity assay as well (Fig. 1B). Therefore, NRRL3 was chosen as the base strain for further optimization towards d-xylitol production in this study.

*A. niger* naturally catabolizes the target compound d-xylitol further to d-xylulose by three dehydrogenases*,* either as the primary reaction via XdhA or as a side reaction via the two dehydrogenases SdhA, and LadA (Fig. 1C). These enzymes primarily catalyze the oxidation of d-xylitol, d-sorbitol, and l-arabitol, respectively [20]. Knocking out all three genes resulted in the accumulation of d-xylitol when d-xylose was consumed [21]. Nevertheless, the presence of LadA is crucial for converting l-arabinose to d-xylitol. We therefore knocked out only the dehydrogenases XdhA and SdhA, resulting in a strain that still consumes some d-xylitol through the side reaction catalyzed by LadA. To reduce the conversion of d-xylitol in another way, we deleted *xkiA*, encoding the d-xylulose kinase, since this enzyme phosphorylates d-xylulose for channeling into the pentose phosphate pathway [21]. When we tested the growth of the triple KO in liquid or solid medium containing d-glucose, d-xylose, l-arabinose, d-sorbitol, or d-xylitol, we observed that growth was strongly reduced in any of the carbon sources except d-glucose (Fig. 1D, E), indicating that pentoses and polyols could indeed not be used as carbon sources by the triple KO strain anymore.

### Evaluation of the influence of strain background and the Fps1 transporter on d-xylitol production from l-arabinose

To streamline the process of extracting the product from the supernatant, the use of a d-xylitol exporter to transport d-xylitol into the surrounding medium was explored. In *S. cerevisiae*, the aquaglyceroporin Fps1, a member of the major intrinsic protein (MIP) family involved in glycerol transport, was found to passively export d-xylitol [39, 40]. A phylogenetic analysis indicated that there is no ortholog of this transporter in the genome of *A. niger* (Supplementary Fig. 1). For this reason, the yeast gene *FPS1* was codon-optimized and cloned to be expressed in *A. niger*.

To enable a constitutively open conformation of the Fps1 transporter, the regulatory region of the protein was deleted [41]. The coding sequence of this version (*FPS1*_open_) was combined with the promoter P*tvdA* and transformed into the *xdhA, sdhA, xkiA* triple KO strain of *A. niger*. We then compared the triple KO strain without Fps1 to the triple KO strain expressing the native or the constitutively open version of Fps1. Furthermore, the open variant of *FPS1* was transformed into the WT background to evaluate the importance of the triple KO for d-xylitol production.

**Fig. 2:**
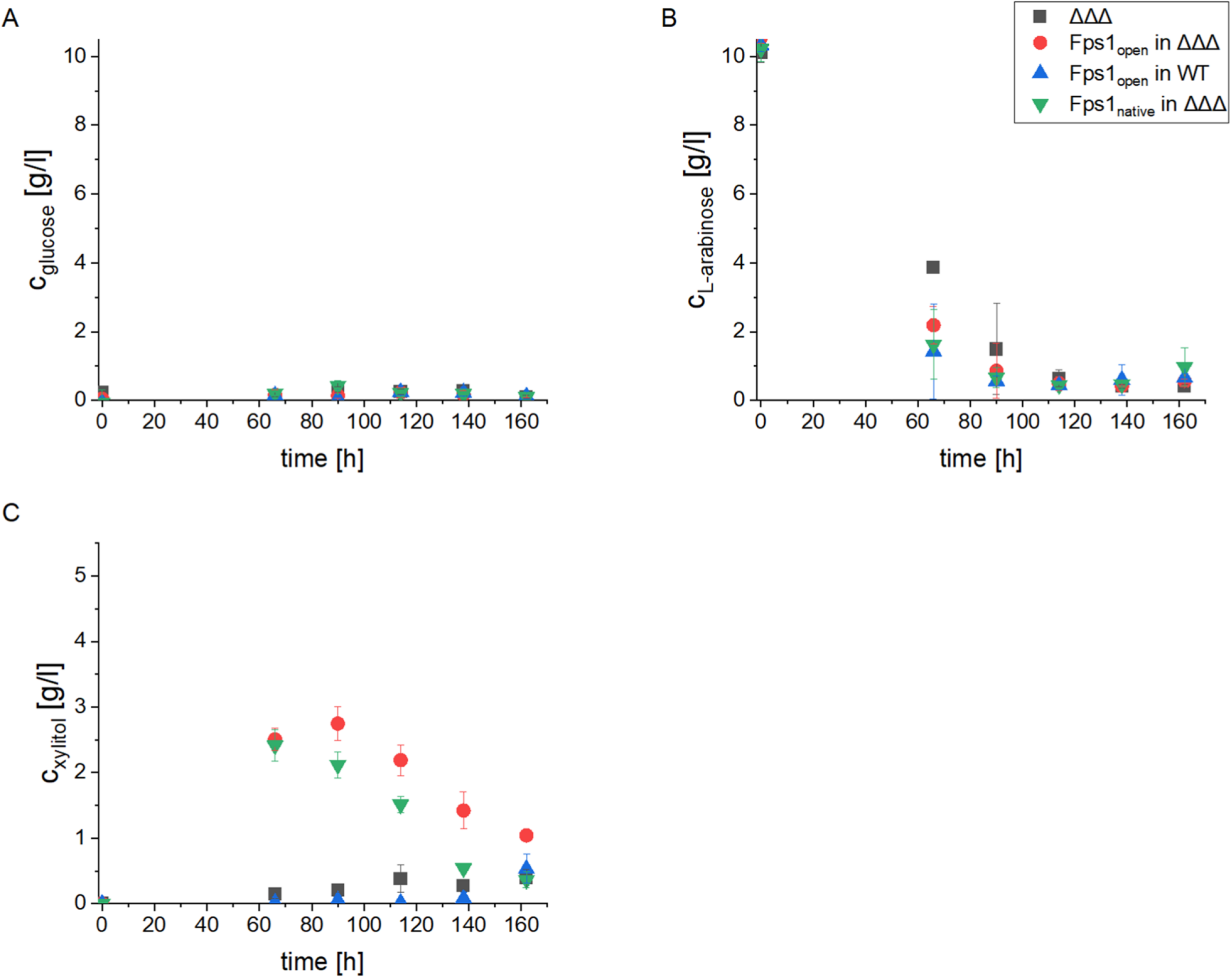
Influence of Fps1 on d-xylitol production from l-arabinose**.** The strains were tested for their d-xylitol production capabilities in 100 ml shake flask experiments. After 99 h of biomass production on 10 g/l of d-glucose (time 0), 10 g/l of l-arabinose was added. After an additional 66, 90, 114, 138, and 162 hs, the supernatant was analyzed by HPLC to measure the concentrations of d-glucose (**A**), l-arabinose (**B**), and d-xylitol (**C**). ΔΔΔ = triple KO of *xdhA, sdhA* and *xkiA.* The Fps1 transporter was expressed under the P*tvdA* promoter for all transporter strains. Data are mean ± standard deviation (n = 4)

After the 1% d-glucose in the pre-culture was completely consumed, 1% l-arabinose was added (t = 0 in Fig. 2). The l-arabinose consumption of the *xdhA, sdhA, xkiA* triple KO strain without the *FPS1* transporter cassette (ΔΔΔ) was delayed compared to the other strains but caught up at 114 h (Fig. 2B). Moreover, d-xylitol production from the supernatant of this strain and from the strain expressing *FPS1*_open_ without the triple KO was very low over the whole time course of the experiment, reaching maximum values of 0.39 g/l d-xylitol at 162 h post l-arabinose addition (Fig. 2C). Comparing the integration of *FPS1*_open_ into the triple KO strain and into the WT strain, a significantly higher production of d-xylitol was observed for the triple KO background in all measurements (p < 0.01, n = 4) with d-xylitol concentrations of 2.75 g/l at 90 h.

The triple KO strain in combination with the *FPS1* transporter variant that is not constitutively open (FPS1_native_), excreted the same amount of d-xylitol as the strain with *FPS1*_open_ (p > 0.05, n = 4) at 66 h. At 90, 114, and 138 h however, the concentrations of d-xylitol in the supernatant were higher for the strain with the open transporter version, reaching up to a difference of 0.88 g/l. The ΔΔΔ *FPS1*_native_ strain nevertheless produced more d-xylitol than either the triple KO strain without transporter or the strain expressing the Fps1 transporter in the WT background (p < 0.01, n = 4). By the end of the experiment (at 162 h), the d-xylitol concentrations in the supernatants of all strains did not differ from each other anymore (p > 0.05, n = 4), indicating post-production degradation.

### Comparison of different *FPS1* expression cassettes

We hypothesized that increasing the abundance of the Fps1 transporter may result in elevated product titers in the supernatant, and would thus be advantageous. To this end, reporter expression cassettes carrying the firefly luciferase gene under control of the P*tvdA*, P*mbfA*, and P*gpdA* from *A. niger* and *A. nidulans* were generated. Moreover, for particularly strong expression of the *FPS1* gene, a novel promoter was generated, combining the native P*gpdA* from *A. niger* with a triplication of the essential gpd-box: P*gpdA*_nig_-3gpd-box [27, 28, 42].

**Fig. 3:**
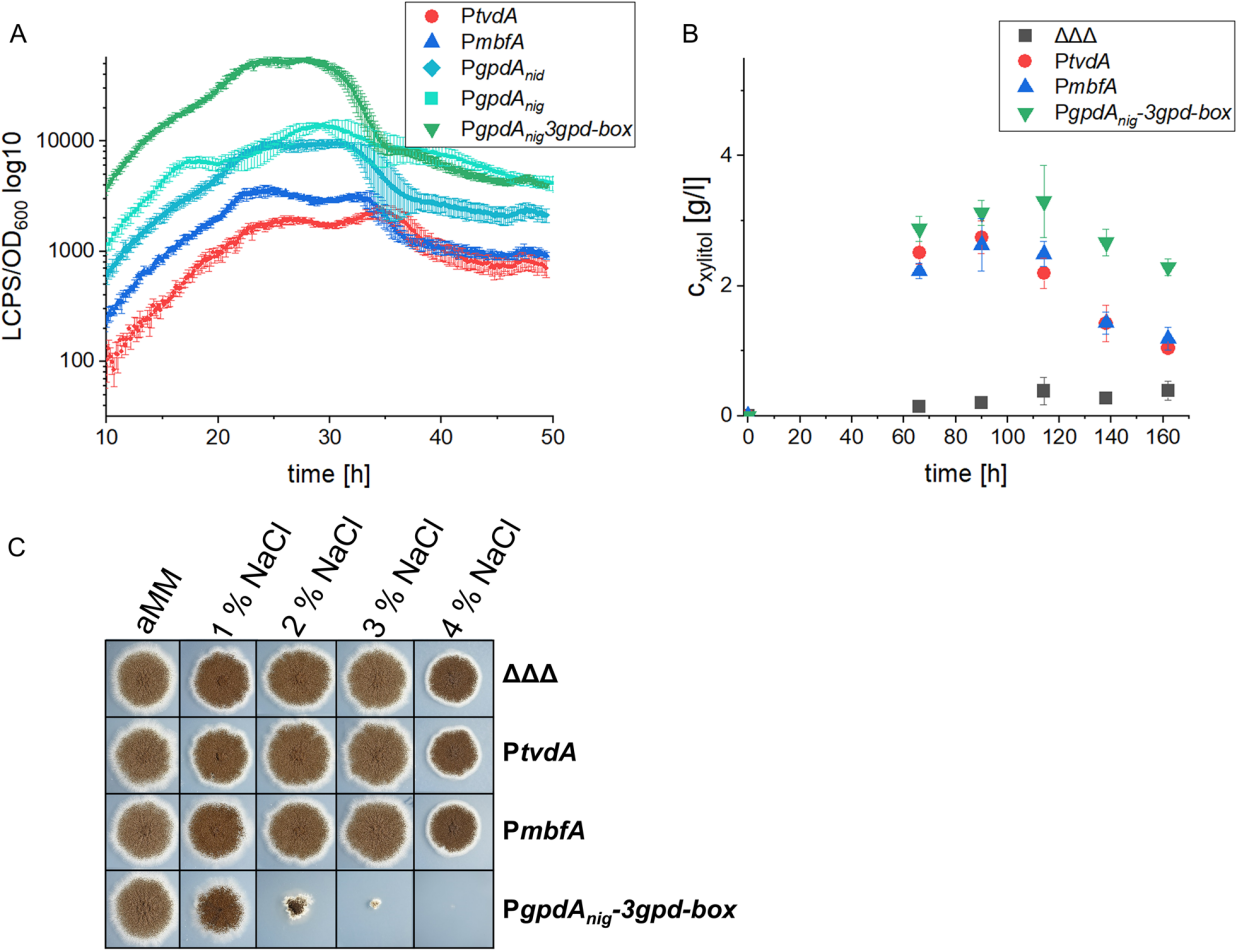
Luminescence of constitutive promoter-reporter-constructs and effects of *FPS1*_*open*_ expression-levels on d-xylitol production and osmotic-stress resistance. **A** Strains expressing a luciferase reporter under the control of several native and artificial promoters were tested in a microtiter plate containing aMM with 2% d-fructose. **B** Xylitol production of the triple KO background strain and strains expressing *FPS1*_*open*_ in ΔΔΔ with three different strengths was tested in 100 ml shake flask experiments containing 100 ml of aMM (same setup as in Fig. 2). Data are mean ± standard deviation (n = 4). ΔΔΔ = triple KO of *xdhA, sdhA* and *xkiA*. **C** The same strains as in (**B**) were compared for growth phenotypes on solid aMM containing 1% d-glucose and NaCl at concentrations 1–4%. Pictures were taken after 6 days

Over the course of 10 to 50 h, the average luminescence values of the strains carrying the P*mbfA,* P*gpdA*_*nid*_, P*gpdA*_*nig*_ or P*gdpA*_*nig*_*3pd-box*-luciferase cassette were 1.7, 4.27, 7.44 and 21.03 times higher than the originally used reference promoter P*tvdA*, respectively (Fig. 3A). Expression of the luciferase reporter under the P*gpdA*_nig_-3gpd-box promoter reached maximum values of 55,231 LCPS/OD_600_, which is 4.02 times higher than the maximum reached in the strain with the *A. niger* P*gpdA* promoter and 15.27 times higher than the strain carrying the P*mbfA* promoter.

Next, the *FPS1*_open_ transporter variant was expressed under the control of the P*mbfA* and P*gpdA*_nig_-3gpd-box promoters to observe the effects of expression strength on d-xylitol production compared to the original design using P*tvdA*-driven expression. The strains expressing P*tvdA*-*FPS1*_open_ and P*mbfA*-*FPS1*_open_ excreted on average 2.75 and 2.63 g/l of d-xylitol at 90 h as their highest yield. P*gpdA*_nig_-3gpd-box-*FPS1*_open_ produced 3.3 g/l of d-xylitol at 114 h (Fig. 3B). The concentration of d-xylitol in the supernatant for P*tvdA*-*FPS1*_open_ and P*mbfA*-*FPS1*_open_ declined more rapidly compared to P*gpdA*_nig_-3gpd-box-*FPS1*_open_, leading to a significant concentration difference at the 114, 138 and 162 h time points post l-arabinose addition (p < 0.05, p < 0.01, p < 0.01, n = 4). P*tvdA*-*FPS1*_open_ and P*mbfA*-*FPS1*_open_ did not differ significantly at any time point.

### Evaluation of transporter expression strains upon osmotic stress

Since Fps1 carries out functions in osmotic tolerance in *S. cerevisiae*, the overexpression of this transporter could lead to impaired fitness upon osmotic stress. For this reason, we decided to subject the strains to different osmotic stress-causing substances and to evaluate the resulting phenotypic effects. Upon adding 2% of NaCl to the media (glucose-containing aMM), only little biomass formation of the strain P*gpdA*_nig_-3gpd-box-*FPS1*_open_ was observed, and no growth was visible at 4% (Fig. 3C, Supplementary Fig. 2), while the triple KO control strain and the two strains with lower *FPS1* expression (P*tvdA*, P*mbfA*) still formed biomass in 4% NaCl. The addition of 1% glycerol to 3% NaCl could partially rescue the *FPS1*_open_ strain under the control of the P*gpdA*_nig_-3gpd-box in liquid culture (Supplementary Fig. 2). Also, d-sorbitol concentrations of 30% inhibited the growth of the P*gpdA*_nig_-3gpd-box-*FPS1*_open_ strain, which was not the case for the other strains. It seemed that the strains P*tvdA*-*FPS1*_open_ and P*mbfA*-*FPS1*_open_ exhibited even better growth compared to the control strain without *FPS1*_open_. To test an osmolyte that cannot be imported, polyethyleneglycol-6000 (PEG) was used. In this case, concentrations of 30% inhibited the growth of the strain with the strongest *FPS1*_open_ expression but not of the other strains. Xylitol addition seemed to inhibit the growth of all strains in a similar fashion, with a strong reduction of biomass being visible at 20%.

### Influence of CreA mutations on d-xylitol production in media containing d-glucose and l-arabinose

**Fig. 4:**
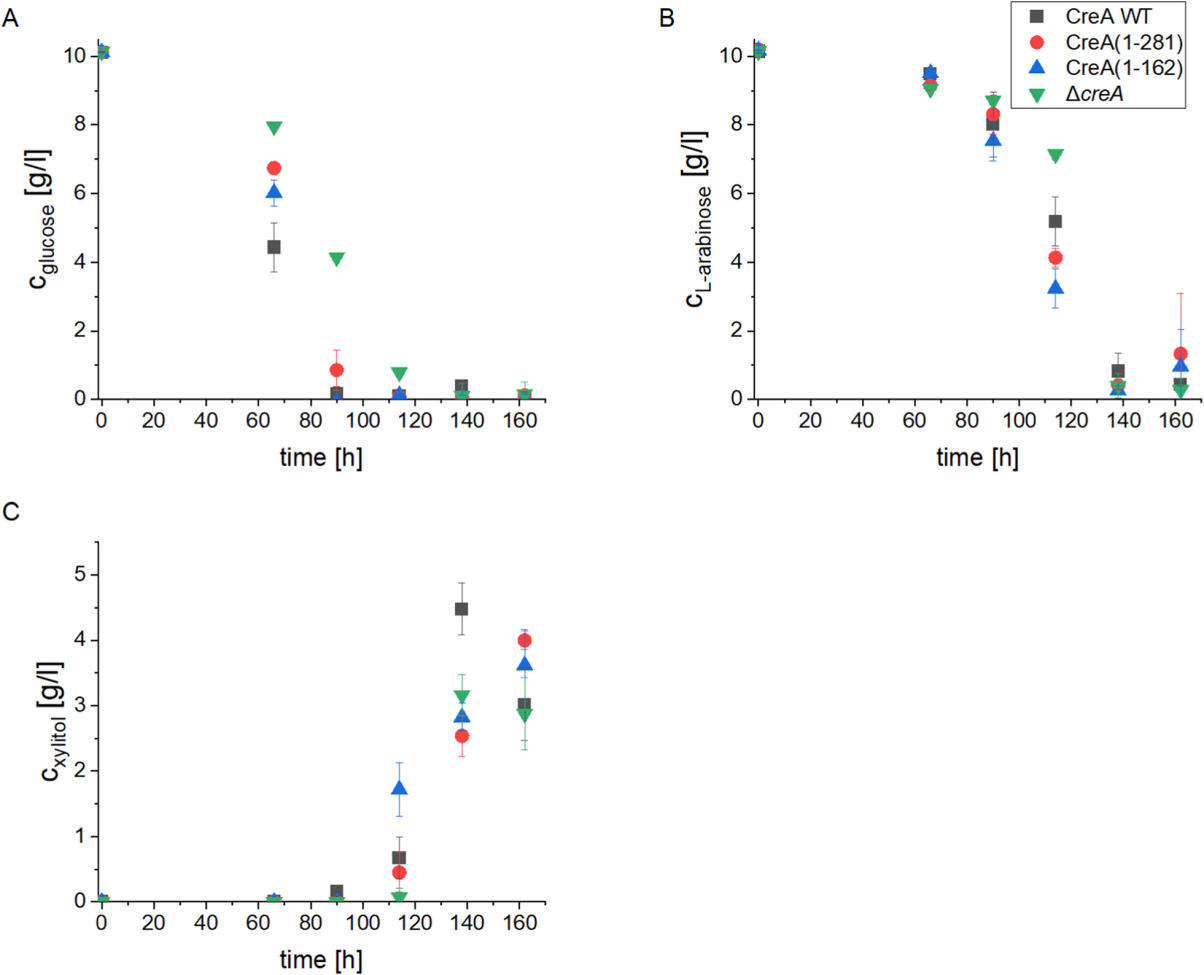
Xylitol production in CreA mutants in the background of the triple KO P*tvdA*-*FPS1*_open_ expression strains. The different CreA truncations/deletions were tested for the production of d-xylitol in media containing 1% l-arabinose + 1% d-glucose and directly inoculated without pre-growth. After 66, 90, 114, 138, and 162 h, the supernatants were analyzed by HPLC to measure the concentrations of d-glucose (**A**), l-arabinose (**B**), and d-xylitol (**C**). Data are mean ± standard deviation (n = 4)

Carbon catabolite repression (CCR) is a regulatory process in various organisms that enables recognition and prioritization of preferred carbon sources, like d-glucose, over other less favored ones [43–45]. In waste streams from sugar production, such as sugar beet pulp, residual d-glucose or sucrose could thus inhibit the production of industrially interesting enzymes and therefore d-xylitol in our studies. Manipulating the main regulator of CCR, CreA, could therefore optimize microbial fermentation processes towards improving the efficiency [46]. CreA truncations and deletions showed to be beneficial in reducing CCR in *A. niger* [35, 47, 48]. To evaluate the effect of CCR on the production of d-xylitol from l-arabinose, CreA mutant strains containing the *FPS1* transporter expressed under P*tvdA* in the triple KO background were inoculated in a medium containing 1% d-glucose and 1% l-arabinose. At 66 h d-glucose was consumed differentially. The WT consumed d-glucose the fastest followed by the strain carrying the shortest CreA truncation (1–162), the longer truncation (1–281), and then the full CreA deletion strain Δ*creA* (Fig. 4A)*.* After 90 h, most of the d-glucose was used up in all strains except the Δ*creA* strain (4.15 g/l d-glucose left). At this time point, the l-arabinose concentration dropped to around 8 g/l in all strains (p > 0.01, n = 4) (Fig. 4B). At time point 114 h, 0.79 g/l of d-glucose was detected in the supernatant of the strain with the Δ*creA* background. For this strain, the l-arabinose concentration was also higher (7.14 g/l) compared to all the other strains. The concentration of l-arabinose in both truncation strains was lower even than in the WT CreA strain, showing a faster consumption of l-arabinose in the strain with the shortest version of CreA. The d-xylitol production of CreA(1–162) was higher at 114 h compared to the WT (Fig. 4C). However, at 138 h the CreA WT strain produced up to 4.48 g/l of d-xylitol, whereas the other strains produced only around 2.5–2.8 g/l of d-xylitol. At time point 162 h, the d-xylitol concentration of the CreA WT strain dropped to the same level as in the other strains (3–3.3 g/l). These results demonstrate that the CreA mutants did not exhibit more efficient d-xylitol production in the tested conditions. However, direct incubation of the xylitol production strain with WT CreA in a medium containing 1% of d-glucose and l-arabinose led to the highest titers of d-xylitol measured so far (4.48 g/l).

## Discussion

Five different *A. niger* strains were tested for their potential to produce endo-arabinanase and α-L-arabinofuranosidase in a medium containing multiple polysaccharides to induce hemicellulolytic enzymes for the generation of l-arabinose as starting C-source for the production of d-xylitol. Endo-arabinanase and α-L-arabinofuranosidase activity varied among strains, with strain NRRL3 emerging as the best overall arabinanase producer. In a previous study, NRRL3 and ATCC 11414 had already been shown to produce high amounts of pectinases [49]. Due to its high arabinanolytic activity, NRRL3 was chosen as a background strain for further optimizations.

The triple KO of *xdhA, sdhA* and *xkiA* was based on previously published work, where *xdhA, sdhA, ladA* and *xkiA* were deleted for d-xylitol production from d-xylose [21]. The KOs were implemented to generate a strain with low d-xylitol degradation. In our approach, we did not knock-out *ladA*, which is needed for d-xylitol synthesis from l-arabinose. Testing the strain on various carbon sources revealed no growth in l-arabinose, d-xylose, d-xylitol and d-sorbitol. Growth in d-glucose showed that the biomass formation of this strain was not overall inhibited by the KO, but the pentoses l-arabinose and d-xylose could not be consumed to generate biomass. By knocking-out *xkiA*, the strain was not able to transform d-xylulose to d-xylulose-5-phosphate. Therefore, it is unable to enter the pentose phosphate pathway, and pentoses cannot be used for growth [21]. As previously described, *sdhA* and *xdhA* are involved in the consumption of d-sorbitol [50]. SdhA and XdhA convert d-sorbitol to d-fructose, which is then further metabolized to generate energy and carbon building blocks [51]. In the triple KO, reduced growth was visible in d-sorbitol, supporting the importance of these enzymes for d-sorbitol consumption and demonstrating that the KOs showed the expected phenotypes.

In the triple KO without a heterologous transporter, the consumption of l-arabinose was delayed compared to the other strains carrying Fps1 transporter variants. Meng et al. demonstrated that d-xylitol accumulates in the cell, which could lead to a reduced metabolic flux towards d-xylitol [21]. This is due to the reversible nature of the reaction converting d-xylitol back to l-xylulose. Consequently, slower consumption of the monosaccharide is observed in comparison to the strains expressing a transporter able to export d-xylitol. In the transporter-expressing strain, it seems that the produced d-xylitol was exported and, therefore, did not inhibit the enzymes of the pentose catabolic pathway. This was also visible when the secreted d-xylitol was measured at 66 h for the strains carrying the combination of the triple KO and the transporter. The WT strain expressing the transporter was not producing significantly higher d-xylitol concentrations compared to the triple KO control. In the WT strain with Fps1, it appeared that the produced d-xylitol was directly consumed and channeled into the pentose phosphate pathway before being exported due to the presence of the enzymes that convert d-xylitol. The strain carrying the native variant of *FPS1* that is not constitutively open produced less d-xylitol after 66 h compared to the *FPS1*_*open*_ variant. The native variant of Fps1 that needs to be actively open in *S. cerevisiae* did not produce such high titers of d-xylitol, letting us assume that the transporter is in an equilibrium between open and closed state in *A. niger*. Therefore, to produce d-xylitol, a combination of the three KOs involved in d-xylitol degradation and an efficient export are necessary.

Karlgren et al. [52] showed that the constitutively open variant of Fps1 transports polyols more efficiently compared to the native version, reinforcing our results [52]. In *S. cerevisiae*, the native variant of Fps1 needs to be open by the regulator of glycerol channel protein 1 and 2 for transport of polyols [53]. The higher d-xylitol titers in the supernatant achieved with the *FPS1*_native_-expressing strain compared to the triple KO without transporter led us to assume that even the native transporter is mostly in an open state. This could be due to differential folding in *A. niger* compared to *S. cerevisiae*, where the transporter needs to be actively open. Alternatively, the regulators that open the transporter are conserved between *S. cerevisiae* and *A. niger.* In the transporter-expressing strains with the triple KO, the d-xylitol values declined after 90 h. The dehydrogenase LadA, which is still able to convert d-xylitol to d-xylulose by its side activity, seems to lower the concentration in the cell, thereby leading to a passive import of d-xylitol into the cytoplasm, creating a sink and converting more d-xylitol over time [20]. Induction of transporter closure of the native Fps1 variant after l-arabinose consumption by salt stress could reduce the consumption of d-xylitol. Furthermore, the oxidation of d-xylitol to d-xylulose is co-factor-dependent [20, 54]. Preventing the oxidation of NADH/H^+^ by generating anoxic conditions could, therefore, reduce the degradation of d-xylitol at the end of the biotransformation, which will nevertheless have to be tested in the future.

To evaluate the influence of the expression strength of *FPS1*, three promoters were initially tested with a luciferase reporter system and then used for expression of *FPS1*. In the literature, it was shown that P*mbfA* expression strength exceeds that of P*tvdA* by tenfold in a β-glucuronidase reporter assay [26]. In our assay over 45 h, the luciferase emission signal was only 1.86 times higher on average. In our study, the luciferase is destabilized by a PEST signal, leading to constant degradation of the enzyme and thus helping to quantify the real-time dynamics of the expression better. Due to the end-point measurement in the β-glucuronidase assay used by Blumhoff et al. an accumulation of the colorimetric product over time could augment the differences between strains. In the same publication, by expressing an aconitate decarboxylase, a 2.5-fold higher production of itaconic acid was measured, which is closer to the factor measured in our assay. However, the production of itaconic acid is impacted by multiple factors, like import of substrate, export of the product, and inhibition of the aconitate decarboxylase by higher concentrations of the product. Therefore, a comparison of the promoters by reporter assays is more suitable.

A 2.96-fold higher expression strength of the luciferase gene was reached by using the *gpdA* promoter from *Aspergillus nidulans* compared to P*mbfA*. This is in contrast to the β-glucuronidase assay performed by Blumhoff et al., in which lower expression was reached compared to P*mbfA*. On the other hand, in the manuscript by Lu et al. [27] a 2.44-fold higher signal of mCherry was measured compared to P*mbfA*, which is quite similar to the results measured here. Furthermore, this work demonstrated a 2.28-fold higher mCherry measurement for the native *A. niger gpdA* promoter compared to the version from *A. nidulans*, which is close to the 1.9-fold higher luminescence measured in our experiments. The gpd-box triplication strategy increased the luciferase signal by another 2.85-fold over the time course, reaching up to 15.27-times higher values compared to the P*gpdA* promoter from *A. niger* at its maximum. Zhang et al. (2018) measured 5.7-times higher values for expression of *xynB* [42].

Expression of the constitutively open version of *FPS1* under the promoters P*tvdA*, P*mbfA*, and P*gpdA*_nig_-3gpd-box provided knowledge of the influence of the expression strength on the d-xylitol production. P*tvdA* and P*mbfA* produced similar amounts of d-xylitol at all time points, in line with their rather similar expression strengths. The strain expressing *FPS1*_open_ under control of the P*gpdA*_nig_-3gpd-box promoter produced the highest amount of d-xylitol and, especially at later time points, the concentration of d-xylitol did not decline as quickly compared to the other *FPS1*_open_-expressing strains.

Testing the triple KO-*FPS1*_open_ expression strains in media with varying osmolytes, the promoter constructs P*tvdA* and P*mbfA* behaved similarly in all the osmolytes except d-sorbitol when compared to the triple KO control strain. It seems that a lower expression of the transporter leads to better growth on rising d-sorbitol concentrations from 30% on. d-sorbitol can be transported by Fps1, and once imported into the cell, it seems to balance out the concentration of the osmolyte, leading to improved resilience [52]. In *S. cerevisiae*, the glycerol-transporting Fps1 closes upon osmotic stress, leading to an accumulation of glycerol in the cell to level out the osmotic potential [39]. Expression of the constitutively open Fps1 variant in *S. cerevisiae* was able to rescue growth on osmolytes in a strain that is not able to produce intercellular glycerol [52].

*FPS1* expressed under the control of the strong P*gpdA*_nig_-3gpd-box led to reduced growth on PEG, d-sorbitol, and NaCl. A general reduction in growth and increased sporulation, indicated by darker colonies, were observed across all strains at 1% NaCl concentration, which is consistent with the findings of Mert and Dizbay [55]. This growth inhibition could be explained by an osmotically induced shift in energy consumption towards the production of conidia. In *A. nidulans* and *A. niger*, the concentration of the polyols glycerol and erythritol rises in the cell upon osmotic stress [56, 57]. Likely, the constitutively open Fps1 leads to the leaking of glycerol and, therefore, reduced osmotolerance [25]. In our study, the addition of 1% glycerol was able to partially rescue the growth of the P*gpdA*_nig_-3gpd-box-*FPS1* strain. Due to the elevated glycerol concentrations outside of the cell, the gradient-controlled efflux of glycerol was weaker, and more glycerol remained inside of the cell, improving the resilience upon salt stress. In the strain that expresses *FPS1* under the P*gpdA*_nig_-3gpd-box promoter, the negative effect of glycerol efflux seemed to be stronger than the positive effect of d-sorbitol influx, and therefore, a reduction of growth was visible at d-sorbitol concentrations of 30% or higher. To minimize the phenotypic side effects of high expression of Fps1, the triple KO strain expressing the P*tvdA*-*FPS1* cassette was used for the follow-up experiments.

For simultaneous consumption of d-glucose and l-arabinose, CreA truncation strains carrying the triple KO and the open version of *FPS1* under the control of P*tvdA* were tested for d-xylitol production and compared to the CreA WT strain. Especially the CreA truncation (1–162) converted l-arabinose more quickly than the WT strain in the presence of d-glucose, demonstrating a reduced CCR in this strain leading to higher d-xylitol concentrations at early time points (114 h). In another study using a CCR luciferase reporter assay, we showed that CreA(1–162) is unable to confer CCR in the presence of 0.5% d-glucose, explaining why the strain switched earlier to l-arabinose utilization than the strain carrying the WT version of CreA [35]. However, since d-xylitol production in the CreA WT strain exceeded the other strains after 138 h, truncation of CreA was overall not beneficial.

Simultaneous consumption of d-glucose supported the production of d-xylitol, and the production strain with the WT version of CreA displayed the highest d-xylitol yields of this study with 4.48 g/l. It needs to be taken into account that d-xylitol production from l-arabinose creates a cofactor imbalance, consuming two NADPH/H^+^ and only one NAD^+^ [22]. This imbalance might be alleviated by d-glucose going through the PPP, which will regenerate NADPH/H^+^ by action of glucose-6-phosphate dehydrogenase and 6-phosphogluconate dehydrogenase, resulting in higher d-xylitol titers compared to sequential growth on d-glucose and l-arabinose. However, increasing simultaneous conversion of d-glucose and l-arabinose in the CreA truncation strain did not further improve d-xylitol production efficiency [58]. In fact, the maximum d-xylitol concentration was even lower in the mutant strains compared to the CreA WT, which could be attributed to slower growth and metabolism in the CreA mutant strains [59].

Overall, a maximal d-xylitol yield of 45% was achieved by combining the triple KO of d-xylitol-catabolizing enzymes with the integration of Fps1 into *A. niger.* In yeasts and *E. coli*, a conversion of l-arabinose to d-xylitol of up to 99% was reached, proving that higher yields are possible [60–62]. Nevertheless, none of these species are efficient plant biomass degraders. Using an engineered *A. niger* strain capable of producing large quantities of plant cell wall-hydrolyzing enzymes could thus be advantageous for generating D-xylitol from complex arabinan-rich plant biomass feedstocks. Our *A. niger* strain is therefore the first filamentous lignocellulolytic fungus engineered for efficient conversion of the pentose L-arabinose to D-xylitol, laying the groundwork for one-pot D-xylitol production from complex biomass.

## Conclusions

By combining the triple KO of Δ*xdhA,* Δ*sdhA* and Δ*xkiA* with a constitutively open version of the aquaglyceroporin Fps1 from *S. cerevisiae*, we were able to generate for the first time a filamentous fungal strain that produces d-xylitol from l-arabinose, with yields of up to 45%. We showed that both the triple KO and the export are necessary to generate high yields of the polyol. We found that constitutively opening of the transporter raises d-xylitol concentration in the supernatant compared to the strain expressing the native version of Fps1. Furthermore, we demonstrated that strong overexpression of *FPS1*_open_ under the control of a newly developed high-expression promoter leads to higher d-xylitol yields compared to weaker expression promoters, but also renders the strain less osmo-tolerant. These findings provide a helpful basis for the production of d-xylitol from l-arabinose or arabinan-containing substrates, offering a more holistic utilization of waste materials like sugar beet pulp.

## Supplementary Information

Supplementary File 1.

Supplementary File 2.

## Data Availability

All data generated or analyzed during this study are included in this published article and its supplementary information files or can be obtained from the corr. Author upon reasonable request.
