# Supplementary material for: Engineering of Aspergillus niger for efficient production of d-xylitol from l-arabinose
Source: Microb Cell Fact. 2024 Oct 5;23:262. doi: 10.1186/s12934-024-02526-7 (PMC11452932; doi:10.1186/s12934-024-02526-7)
Supplement: Supplementary file 1 [file 12934_2024_2526_MOESM1_ESM.pdf]

Microbial Cell Factories

Engineering of *Aspergillus niger* for efficient production of D-xylitol from L-arabinose

Marcel Rüllke[1], Veronika Schönrock[1], Kevin Schmitz[1], Mislav Oreb[2], Elisabeth Tamayo[1], J. Philipp Benz[1]

[1] Fungal Biotechnology in Wood Science, Holzforschung München, TUM School of Life Sciences, Technical University of Munich, 85354 Freising, Germany.

[2] Faculty of Biological Sciences, Institute of Molecular Biosciences, Goethe University Frankfurt, 60438, Frankfurt am Main, Germany.

Supplementary figures

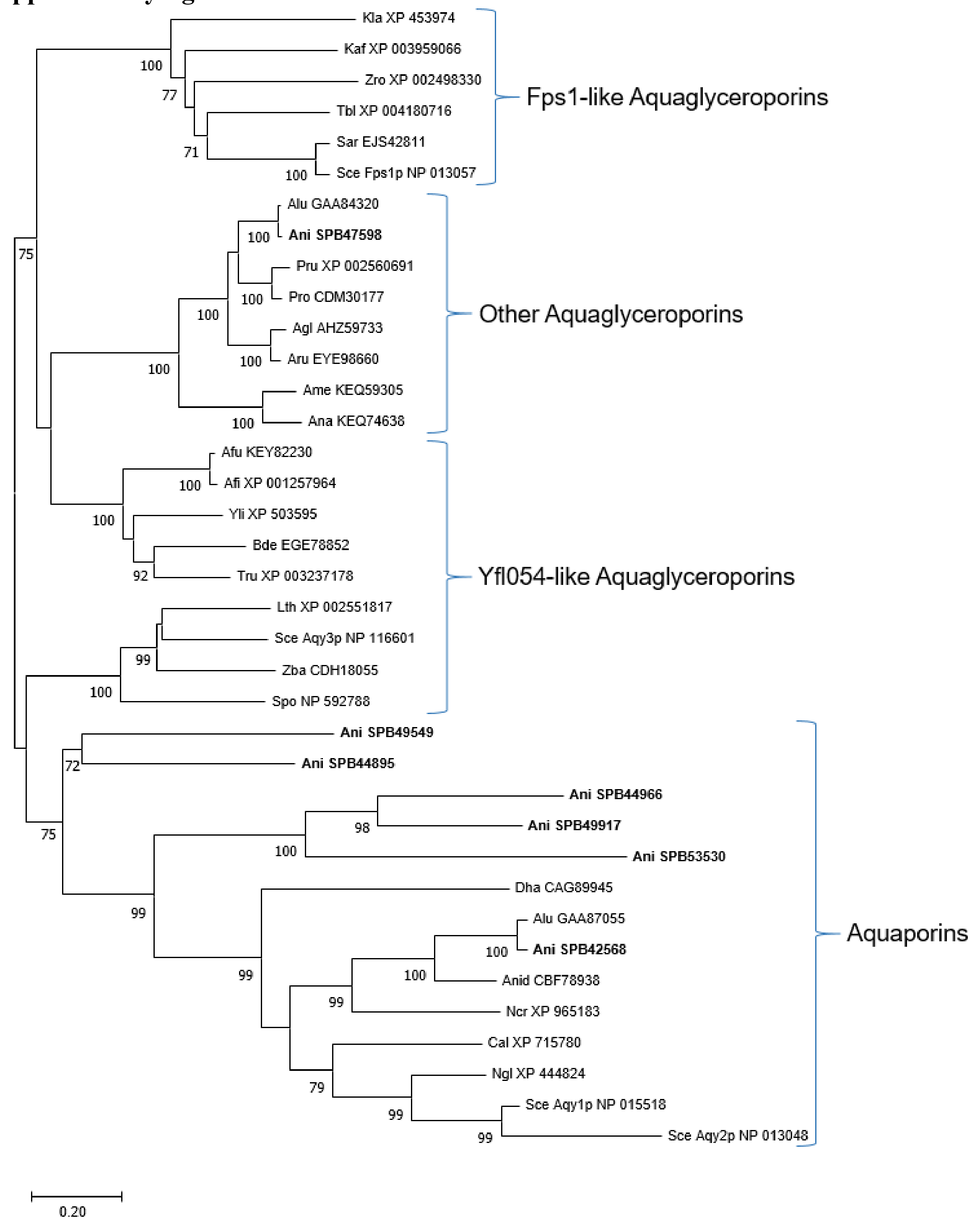

Supplementary Fig. 1 Unrooted Neighbor-Joining tree of the AQPs in *Aspergillus niger* N402. *A. niger* does not contain any Fps1-like aquaglyceroporin in its genome. Organisms: Afi, *Aspergillus fischeri*; Afu, *Aspergillus fumigatus*; Agl, *Aspergillus glaucus*; Alu, *Aspergillus luchuensis*; Ame, *Aureobasidium melanogenum*; Ana, *Aureobasidium namibiae*; Anid, *Aspergillus nidulans*; Ani, *Aspergillus niger*; Aru, *Aspergillus ruber*; Bde, *Blastomyces dermatitidis*; Cal, *Candida albicans*; Dha, *Debaryomyces hansenii*; Kaf, *Kazachstania africana*; Kla, *Kluyveromyces lactis*; Lth, *Lachancea thermotolerans*; Ncr, *Neurospora crassa*; Ngl, *Nakaseomyces glabratus*; Pro, *Penicillium roqueforti*; Pru, *Penicillium rubens*; Sar, *Saccharomyces arboricola*; Sce, *Saccharomyces cerevisiae*; Spo, *Schizosaccharomyces pombe*; Tbl, *Tetrapisispora blattae*; Tde, *Torulaspora delbrueckii*; Tru, *Trichophyton rubrum*; Yli, *Yarrowia lipolytica*; Zba, *Zygosaccharomyces bailii*; Zro, *Zygosaccharomyces rouxii*. *A. niger* AQPs are emphasized in bold. Protein NCBI identification numbers are indicated. Bootstrap values above 70 are indicated.

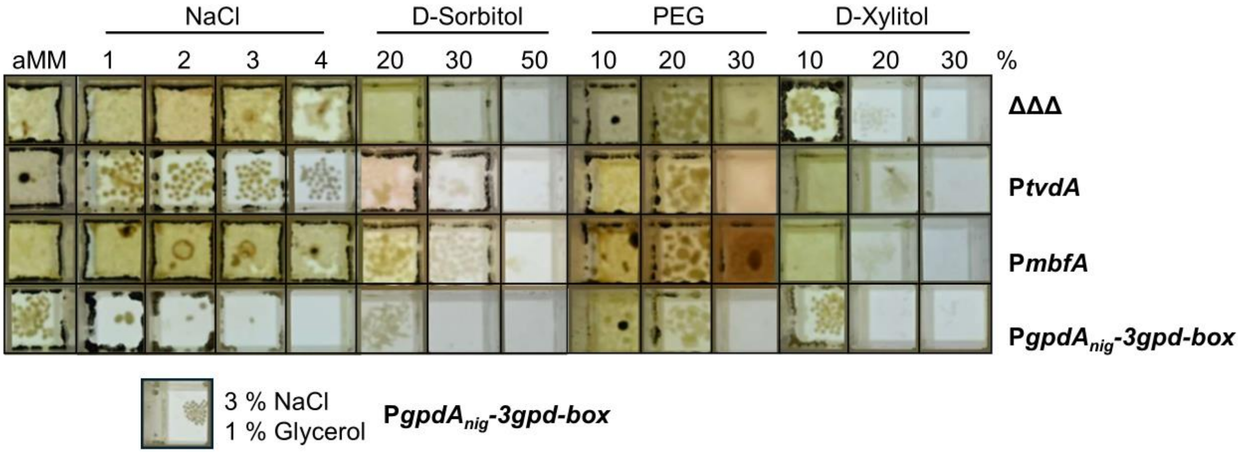

Supplementary Fig. 2 The triple KO of *xdhA*, *sdhA* and *xkiA* (ΔΔΔ) was compared to the *FPS1*$_{open}$ expression strains for growth phenotypes in aMM containing 1 % D-glucose and four osmolytes using a gradient of concentrations. Pictures were taken 6 days after incubation at 30 °C in the dark.
